# The Use of Transcranial Magnetic Stimulation in Attention Optimization Research: A Review from Basic Theory to Findings in Attention-Deficit/Hyperactivity Disorder and Depression

**DOI:** 10.3390/life14030329

**Published:** 2024-02-29

**Authors:** Chiahui Yen, Ethan P. Valentine, Ming-Chang Chiang

**Affiliations:** 1 Department of International Business, Ming Chuan University, Taipei 111, Taiwan; chyen@mail.mcu.edu.tw; 2 Department of Addiction Studies, Psychology, and Social Work, Minot State University, Minot, ND 58707, USA; ethan.valentine@minotstateu.edu; 3 Department of Life Science, College of Science and Engineering, Fu Jen Catholic University, New Taipei City 242, Taiwan

**Keywords:** transcranial magnetic stimulation, attention, attention-deficit/hyperactivity disorder, depression

## Abstract

This review explores the pivotal role of attention in everyday life, emphasizing the significance of studying attention-related brain functions. We delve into the development of methodologies for investigating attention and highlight the crucial role of brain neuroimaging and transcranial magnetic stimulation (TMS) in advancing attention research. Attention optimization theory is introduced to elucidate the neural basis of attention, identifying key brain regions and neural circuits involved in attention processes. The theory further explores neuroplasticity, shedding light on how the brain dynamically adapts and changes to optimize attention. A comprehensive overview of TMS is provided, elucidating the principles and applications of this technique in affecting brain activity through magnetic field stimulation. The application of TMS in attention research is discussed, outlining how it can be employed to regulate attention networks. The clinical applications of TMS are explored in attention-deficit/hyperactivity disorder (ADHD) and depression. TMS emerges as an effective clinical treatment for ADHD, showcasing its potential in addressing attention-related disorders. Additionally, the paper emphasizes the efficacy of TMS technology as a method for regulating depression, further underlining the versatility and therapeutic potential of TMS in clinical settings. In conclusion, this review underscores the interdisciplinary approach to attention research, integrating neuroimaging, neuroplasticity, and TMS. The presented findings contribute to our understanding of attention mechanisms and highlight the promising clinical applications of TMS in addressing attention-related disorders. This synthesis of theoretical and practical insights aims to propel further advancements in attention research and its therapeutic applications.

## 1. Introduction

### 1.1. The Importance of Attention in Everyday Life

Attention is a fundamental cognitive process that plays a crucial role in everyday life [1]. It is the ability to selectively focus on specific stimuli, information, or tasks while filtering out irrelevant or distracting information [2]. Attentional processes contribute directly to a multitude of cognitive skills. For instance, attention is essential for effective task performance [3]. Whether studying, working, driving, or even engaging in a simple conversation, directing your attention to relevant cues helps you process information accurately and make informed decisions. Attention is also a prerequisite for learning and memory formation, as information that is attended to is more likely to be encoded and stored in memory for future retrieval [4]. Without attention, new information can easily be overlooked and forgotten. In addition, when faced with complex problems, attention enables us to break down the problem into its components, focus on relevant details, and identify potential solutions. Attention further contributes to maintaining personal safety and situational awareness, communicating effectively via attentive listening, and managing competing tasks/deadlines. Creativity and innovation also depend on attentional processes, as attention improves the ability to focus on novel ideas and perspectives, enabling divergent thought. Attention switching allows us to explore different aspects of a problem or situation, leading to fresh insights. Attention also plays a role in emotional regulation, as directing attention away from negative or distressing stimuli (and towards positive or calming ones) can lead to an improved emotional state [5]. Effective time management relies on allocating attention to tasks based on their priority and urgency. Proper attention management can enhance productivity and reduce stress. Even everyday choices benefit from attentional processes as we choose to focus on relevant information or available options. Attention is the cognitive mechanism that allows us to engage with the world around us [6], make sense of our experiences, and navigate our daily lives. Its importance cannot be overstated, as it influences nearly every aspect of our cognitive functioning and interactions with the environment. This review provides relevant keywords for conducting a literature search on neuroimaging and its applications. These keywords include TMS, attention, ADHD, depression, brain function, neurological diseases, treatment strategies, and terms related to the biological cellular components, cellular pathways, synaptopathies, synaptic plasticity, and neurobiology. Describing the databases and sources searched, such as PubMed, Scopus, or other relevant platforms, is essential when conducting a literature search. Additionally, it is necessary to provide information on the search terms and keywords used to identify appropriate research. Boolean operators such as ‘AND’, ‘OR’, and ‘NOT’ can refine searches and find relevant articles. Additionally, it is essential to consider using specific terms related to topics of interest, such as particular brain regions, cognitive processes, or specific methods and techniques.

This could be particularly relevant to how external stimuli and the passive perception of sensory influx during speech-related activities influence the brain’s attention system. Referencing relevant studies, such as Callan et al.’s work on song and speech, would further support and substantiate the discussion [7]. Including these elements would contribute to a more nuanced exploration of attention processes in the context of sensory perception and action. The modulation of the brain’s attention system through passive perception of sensory influx refers to the influence of sensory input on attentional processes without active engagement or intentional focus [8]. In this context, sensory information from the environment, such as auditory or visual stimuli, can impact attentional mechanisms even when an individual is not consciously directing their attention toward that specific input [9]. Bottom–up processing is a type of attention modulation that is often associated with sensory perception, where the salience or significance of environmental stimuli drives attentional shifts. This contrasts with top–down processing, where goals and intentions guide attention. The brain’s attentional system, including the prefrontal and parietal cortex regions, can also be influenced by incoming sensory input [10]. Neural circuits associated with attention may respond to salient stimuli, leading to changes in cognitive processing. In speech perception, passive auditory perception plays a crucial role. The brain automatically processes speech sounds, even when individuals are not actively listening or producing speech. This highlights the interconnectedness of perception and action in the neural mechanisms underlying attention. Understanding how the brain’s attention system is modulated through passive perception contributes to the broader understanding of cognitive flexibility, which has substantive implications for the broader research literature [11]. The brain’s ability to adapt to and prioritize different sensory inputs, even without conscious direction, is essential for efficient information processing. In summary, the modulation of the brain’s attention system through passive perception of sensory influx underscores the dynamic and automatic nature of attentional processes, shedding light on how the brain responds to environmental stimuli without intentional focus. This concept is pertinent to various domains, including speech perception, cognitive flexibility, and clinical neuroscience.

### 1.2. The Importance of Brain Neuroimaging and TMS in Attention Research

Brain neuroimaging and TMS have emerged as crucial tools in attention research [12,13]. Neuroimaging techniques like functional magnetic resonance imaging (fMRI) [14] and positron emission tomography (PET) allow us to visualize and understand the brain regions and networks involved in attention processes. By observing changes in neural activity, connectivity, and blood flow, these methods offer insights into the intricate mechanisms underlying attention. On the other hand, TMS provides a noninvasive means of modulating brain activity [15,16]. TMS can temporarily enhance or disrupt neural functioning by applying magnetic fields to specific brain regions, offering a unique opportunity to investigate causal relationships between brain areas and attentional functions [17]. This technique enables researchers to explore how specific brain regions contribute to attention control, enhancing our understanding of attention-related disorders and potential therapeutic interventions [18]. Combining brain neuroimaging and TMS offers a comprehensive approach to studying attention [19]. Neuroimaging provides a map of brain activity, while TMS allows manipulation of this activity, revealing how changes in neural circuits impact attentional processes [20]. The integration of these techniques holds promise for unveiling the complexities of attentional networks, shedding light on their dysfunctions in disorders like ADHD and depression [21]. This holistic approach could lead to more effective diagnostic tools and targeted interventions for individuals facing attention-related challenges.

While fMRI is a commonly used neuroimaging technique, optical methods such as functional near-infrared spectroscopy (fNIRS) or diffuse optical imaging (DOI) offer different advantages and considerations, especially when paired with TMS [22,23]. fNIRS measures changes in blood oxygenation similar to fMRI. DOI is another optical method that measures light scattering and tissue absorption changes. This technique provides information about changes in blood flow and oxygenation. Some key points to consider in explaining the specifics of optical methods in the context of TMS are as follows. Temporal resolution: Optical methods, particularly fNIRS, provide a higher temporal resolution than fMRI [24]. This is crucial when investigating the dynamic changes in brain activity induced by TMS. Spatial resolution: fMRI typically offers better spatial resolution than optical methods, but fNIRS can still provide sufficient spatial information, especially in regions close to the scalp. Combining TMS and optical methods can help capture spatial and temporal dynamics simultaneously. Direct compatibility with TMS: Unlike other neuroimaging techniques, optical methods are less sensitive to the magnetic fields generated by TMS. This makes them more directly compatible, allowing concurrent TMS-fNIRS or TMS-DOI experiments without significant interference [25]. Depth of penetration: Optical methods are beneficial for investigating cortical activity, making them well suited for TMS studies, which primarily affect surface-level cortical regions. Addressing these points can offer a more comprehensive understanding of the role of optical neuroimaging techniques when employed in TMS studies, thus enriching the discussion on the methodologies used in attention research.

## 2. Attention Optimization Theory

### 2.1. Neural Basis of Attention: Brain Regions and Neural Circuits Related to Attention

The neural basis of attention involves a complex interplay of various brain regions and neural circuits that work together to facilitate attentional processes [26,27]. Some of the critical brain regions and circuits associated with attention include the following. The prefrontal cortex (PFC): this region is crucial for executive functions, including sustained attention, working memory, and cognitive control. The dorsolateral prefrontal cortex (DLPFC): this region is involved in top–down control of attention, guiding the focus of attention based on goals and intentions [28]. Parietal lobe: The posterior parietal cortex (PPC) plays a role in spatial attention and integrating sensory information. It helps in orienting attention to relevant stimuli and filtering out distractions. Temporal lobe: the superior temporal sulcus (STS) is involved in social attention, recognizing faces, and interpreting social cues [29]. Visual cortex: the primary visual cortex (V1) processes basic visual information, while higher visual areas, such as the ventral and dorsal streams, are responsible for object recognition and spatial processing, respectively [30]. Anterior cingulate cortex (ACC): the ACC is implicated in detecting conflicts and monitoring for errors, contributing to selective attention and response inhibition [31]. Thalamus: this structure serves as a relay station for sensory information and facilitates and directs sensory input to relevant brain regions. Reticular activating system (RAS): this network of neurons in the brainstem helps regulate wakefulness, alertness, and arousal levels, impacting the overall attentional state. Basal ganglia: The basal ganglia modulate attention through their influence on motor functions and cognitive control. They contribute to action selection and inhibition. Default mode network (DMN): This network is active when the mind is at rest and less engaged in external tasks. It becomes less active during focused attention, indicating its role in mind-wandering and self-referential thinking. Salience network: Comprised of the insula and anterior cingulate cortex, this network helps detect relevant stimuli and determine what to pay attention to. These brain regions are interconnected through neural pathways, forming intricate circuits that enable attention processes [32]. Neural communication within and between these regions enables us to selectively process sensory information, maintain focus, switch attention between tasks, and filter out irrelevant stimuli [33]. Dysfunction in these circuits can contribute to attention-related disorders such as ADHD or contribute to attention impairments in conditions like depression [34,35].

### 2.2. Neuroplasticity: Explores How the Brain Adapts and Changes to Optimize Attention

Neuroplasticity, or brain plasticity, refers to the brain’s remarkable ability to adapt and change its structure and functioning in response to experiences, learning, and environmental demands [11]. In the context of attention optimization, neuroplasticity plays a pivotal role in shaping and refining the brain’s attentional processes. Here is how neuroplasticity contributes to optimizing attention. Experience-dependent changes: Neuroplasticity enables the brain to rewire itself based on experiences. As individuals engage in tasks that require focused attention, the neural circuits associated with attention-related brain regions strengthen through repeated use [36]. This results in improved efficiency in processing relevant information. Learning and training: Practice and learning drive neuroplastic changes. Activities that demand sustained attention, like meditation or cognitive training exercises, can induce structural and functional alterations in key attention-related areas [37]. These changes enhance attentional control and allocation. Synaptic strengthening: Neuroplasticity involves strengthening the connections between neurons, known as synapses. Intense focus and engagement increase synaptic strength in attention-related pathways, making signal transmission more efficient. Neural network adaptations: Neuroplasticity does not just impact individual neurons; it also influences entire neural networks. Through repeated attention-demanding tasks, networks involved in attentional processes become more finely tuned and interconnected, optimizing their collaborative efforts. Neurotransmitter changes: Neuroplasticity can affect neurotransmitter systems that regulate attention. Dopamine, for example, plays a role in reward and motivation, and its release can be modulated by sustained attention tasks, contributing to improved attentional performance. Environmental enrichment: exposure to enriched environments with varied sensory and cognitive stimuli fosters neuroplasticity, encourages the growth of new neural connections, and supports cognitive functions, including attention [38]. Recovery from injury: Neuroplasticity is crucial for postinjury recovery. After brain damage, other regions scan, adapt, and take over functions from the affected area, aiding in attention recovery. Understanding the mechanisms of neuroplasticity offers insights into how interventions can be designed to optimize attention [39]. Training programs, therapies, and lifestyle changes encouraging focused and sustained attention can harness neuroplasticity to enhance attentional abilities. Furthermore, individuals with attention deficits can benefit from targeted interventions that leverage neuroplasticity to rewire attention-related neural networks [40]. Overall, exploring neuroplasticity provides a pathway to unlocking the brain’s potential for improving attention and other cognitive functions.

## 3. Basic Theory of Transcranial Magnetic Stimulation

### 3.1. Principles and Applications: Explain How TMS Affects Brain Activity through Magnetic Field Stimulation

TMS is a noninvasive technique that involves the application of magnetic fields to the brain to modulate neural activity [41,42,43,44]. The basic theory underlying TMS involves electromagnetic induction and its effects on neuronal firing [45,46]. Here is an overview of the basic theory of TMS. Electromagnetic induction: TMS utilizes Faraday’s law of electromagnetic induction [47]. When a rapidly changing magnetic field is applied near a conductor, such as the brain’s neural tissue, it induces an electric field within the tissue. This electric field can depolarize neurons, triggering action potentials and influencing their activity. Neuronal activation: the induced electric field generated by TMS can lead to neuronal depolarization if it reaches a certain threshold [12]. Neurons perpendicular to the magnetic field are most affected, and if the stimulation is strong enough, it can cause neurons to fire action potentials. Neuronal plasticity: Repetitive TMS (rTMS) protocols can induce longer-lasting effects on neural circuits. Depending on the stimulation frequency, rTMS can either increase or decrease the excitability of the stimulated brain region [48]. High-frequency rTMS can enhance synaptic strength and excitability, potentially leading to long-term potentiation (LTP)—a mechanism associated with learning and memory [49]. Spatial and temporal specificity: The effects of TMS depend on factors such as the intensity, frequency, and duration of the magnetic pulses. Additionally, the specific brain region the TMS coil targets determines which neural circuits are influenced. Coil design and positioning contribute to the precision of stimulation, allowing researchers to target specific brain regions. Therapeutic applications: TMS is used in various therapeutic applications, including the treatment of neuropsychiatric disorders like depression [50]. Optical neuroimaging involves using various optical techniques to visualize and monitor brain activity. When applied to TMS, optical methods could explore psychiatric disorders [50]. Optical neuroimaging involves using various optical techniques to visualize and monitor brain activity. When applied to TMS, optical methods can understand the effects of TMS on brain function. In the context of TMS, fNIRS can help assess how TMS modulates cerebral blood flow and oxygenation in specific brain regions [19]. DOI can be applied to study the effects of TMS on neural activity by monitoring hemodynamic responses in the brain. Applying optical neuroimaging to TMS studies allows for a more comprehensive understanding of how TMS affects brain function, both in immediate changes in neural activity and potential long-term effects on brain connectivity. By modulating brain activity in specific regions, TMS can help normalize abnormal neural circuits associated with these disorders [51]. Investigative tool: TMS serves as a valuable tool for probing brain–behavior relationships. By temporarily disrupting or enhancing brain regions, researchers can infer the roles of these regions in various cognitive processes [17]. In summary, the basic theory of TMS involves inducing electric fields in the brain’s neural tissue by applying rapidly changing magnetic fields [52,53,54]. This electric field can alter neuronal activity, producing immediate and potentially long-lasting effects on neural circuits [55]. TMS offers insights into brain function and is a therapeutic option for various neurological and psychiatric conditions [52,56,57].

### 3.2. Application of TMS in Attention Research: Discuss How TMS Can Be Applied to Regulate Attention Networks

TMS holds promise as a tool for regulating attention networks by modulating specific brain regions involved in attention processes [58,59]. Here is how TMS can be applied in attention research. Targeted brain regions: TMS allows researchers to selectively target and stimulate or inhibit specific brain regions associated with attention. Brain regions such as the prefrontal, parietal, and anterior cingulate cortex are crucial for attention control [60]. Researchers can temporarily alter their activity by applying TMS to these areas and studying the effects on attentional performance. Enhancing attention networks: High-frequency repetitive TMS (rTMS) can increase the excitability of targeted brain regions. When applied to attention-related areas, this protocol can potentially enhance attentional functions. For example, stimulating the dorsolateral prefrontal cortex (DLPFC) may improve sustained attention and cognitive control [61]. Cognitive training and rehabilitation: TMS could be integrated into cognitive training protocols to improve attention. Combining TMS with cognitive exercises may enhance the neuroplastic changes associated with attention networks, potentially leading to longer-lasting improvements in attentional abilities [62]. Clinical interventions: Attention-related disorders such as ADHD involve dysfunctions in attention networks. TMS might offer a novel therapeutic avenue by directly targeting and modulating these networks [17,58]. Combining TMS with neuroimaging: Integrating TMS with functional neuroimaging techniques like fMRI allows researchers to monitor real-time changes in brain activity during TMS sessions [20,63]. This combination provides a comprehensive understanding of the dynamic interactions within attention networks. While applying TMS in attention research is promising, it is essential to recognize that TMS effects can be transient and highly context-dependent [64]. Additionally, individual variability and potential risks must be carefully addressed when applying TMS techniques [53]. Nonetheless, TMS offers a powerful tool for investigating and potentially modulating attention networks, shedding light on their functioning and providing insights into attention-related disorders and interventions [54].

### 3.3. Molecular and Cellular Mechanisms Underlying TMS Effects on the Brain

Understanding TMS’s biological mechanisms is crucial for comprehending its impact on the brain. Primary mechanism: TMS uses a coil that generates a rapidly changing magnetic field. This magnetic field induces electrical currents in the underlying neural tissue, particularly in the cerebral cortex [12,65]. Neuronal activation: When the magnetic field penetrates the skull and reaches the brain, it induces electric currents in neurons. This electrical stimulation depolarizes neurons, leading to action potentials. Excitation and inhibition: the effects of TMS can lead to either excitation or inhibition of neuronal activity, depending on factors such as the frequency and intensity of the magnetic pulses [45]. High-frequency TMS increases neuronal activity, while low-frequency TMS may decrease it. Focal stimulation: TMS allows for focal stimulation of specific brain regions depending on the coil’s placement. Effects on synaptic plasticity: TMS has been shown to induce changes in synaptic plasticity, influencing the strength of connections between neurons [49]. This can lead to both short-term and long-term effects on neural circuits. Brain regions targeted: The impact of TMS is most prominent in the regions directly beneath the coil. Commonly targeted areas include the motor cortex, prefrontal cortex, and other regions implicated in various cognitive functions [12]. Connectivity effects: TMS affects the directly stimulated region and influences connected neural networks. This network-level impact is crucial for understanding how TMS can modulate broader brain functions. In a clinical context, TMS is applied therapeutically, with repetitive sessions potentially leading to longer-lasting changes in neural activity [66]. In summary, TMS works by inducing electrical currents in the brain through a changing magnetic field, leading to neuronal activation or inhibition. The technique’s precision allows for targeted stimulation of specific brain regions, and its effects extend to influencing synaptic plasticity and broader neural networks. Understanding these biological mechanisms is essential for both research and therapeutic applications of TMS in modulating brain function.

**Figure 1:**
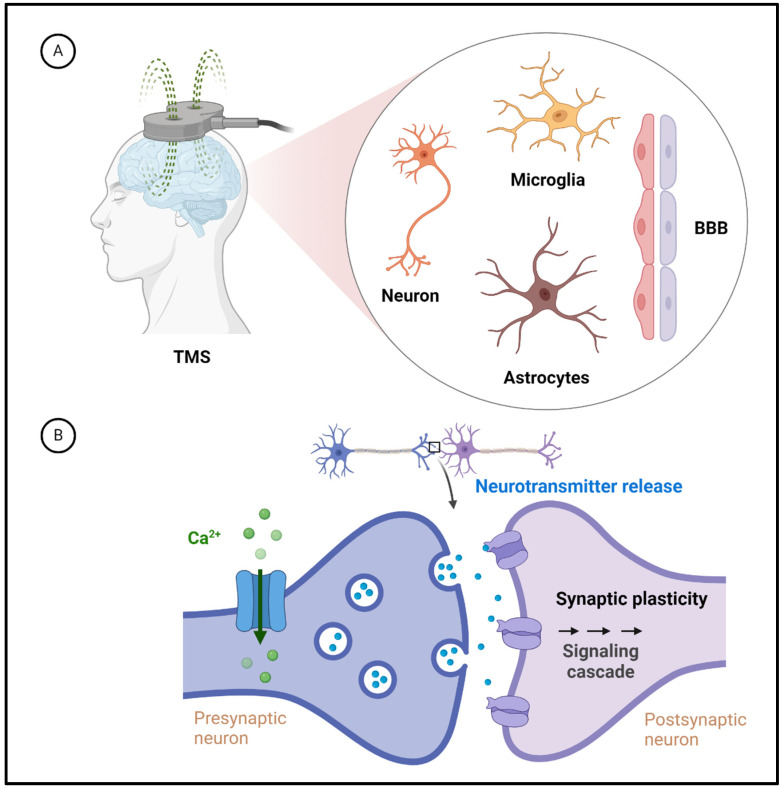
TMS affects neuroplasticity in neurons, astrocytes, microglia, and the BBB. (**A**) TMS affects neurons mainly by inducing current in the neuronal membrane, leading to depolarization and action potential generation. This involves the observed changes in brain activity and function. TMS also affects stellate cells, regulating synaptic transmission. Microglia are immune cells in the brain that may respond indirectly to TMS-induced neural activity and connectivity changes. TMS may affect BBB permeability, causing effects on neural tissue. (**B**) TMS affects synaptic plasticity by changing the strength of neuronal connections, thereby affecting neural circuits. Magnetic fields induce changes in ion flow, particularly calcium ion flow, which are critical for neurotransmitter release and overall neuronal communication. TMS activates neurons, increases neurotransmitter release, modulates synaptic transmission, and affects brain function. It also involves directly stimulated neurons and interconnected neural networks, altering synaptic connections and inducing neuroplasticity–structural and functional changes in synapses. The blue dots in the picture represent neurotransmitters. Figure 1 was created with BioRender.

The biological cellular components and cellular and molecular mechanisms affected by TMS provide a more detailed understanding of its effects on the brain (Figure 1). Neuronal Activation: The primary target of TMS is the neurons in the cerebral cortex. When the rapidly changing magnetic field generated by the TMS coil reaches the brain, it induces electrical currents in neurons, leading to depolarization and the generation of action potentials [45]. Ion Channels: TMS is believed to influence ion channels on the neuronal membrane. The magnetic field induces changes in the flow of ions, particularly calcium ions, across the neuronal membrane [67,68]. Calcium influx is a crucial step in initiating neurotransmitter release and the overall process of neuronal communication. Neurotransmitter Release: Activating neurons through TMS may increase neurotransmitter release [69]. This effect can modulate synaptic transmission and contribute to the observed changes in brain function. Connectivity effects: TMS influences the directly stimulated neurons and affects interconnected neural networks [70]. Modifying synaptic connections between neurons contributes to the broader effects on brain function. Blood–brain barrier (BBB): TMS is generally considered a noninvasive technique, and its effects are primarily localized to the neural tissue beneath the coil. However, changes in neural activity induced by TMS could potentially trigger secondary effects that may influence BBB permeability [71]. Cellular transporters: The specific cellular components affected by TMS include various ion channels, receptors, and transporters. TMS may influence neurotransmitter transporters’ activity, impacting neurotransmitter reuptake and their concentration in the synaptic cleft [15]. Neuroplasticity: TMS-induced changes in neural activity can lead to neuroplasticity, involving structural and functional alterations in synapses [72,73]. In summary, while TMS primarily targets neurons in the cerebral cortex and influences ion channels and neurotransmitter release, the precise molecular and cellular mechanisms are complex and multifaceted.

The specific cell types (Figure 1) affected by TMS provide a more comprehensive understanding of its cellular-level impact on the brain. Neurons are the primary cellular targets of TMS [12,74]. The magnetic field induces electrical currents in neuronal membranes, leading to depolarization and the generation of action potentials. The effects on neurons contribute to the observed brain activity and function changes. Astrocytes, which are glial cells, play a crucial role in supporting neuronal function. TMS has been shown to influence astrocytic calcium signaling. Changes in astrocytic activity could have modulatory effects on synaptic transmission and contribute to the overall impact of TMS on neural networks [75]. Microglia are immune cells in the brain responsible for surveillance and response to neural activity; changes in neural activity and connectivity induced by TMS may indirectly influence microglial responses. Activation patterns: The specific areas of the brain affected by TMS depend on the placement of the coil, targeting various cortical regions, such as the motor cortex, prefrontal cortex, or parietal cortex. Different brain regions may show varying responses to TMS, leading to diverse physiological and cognitive effects [12]. Connectivity and network effects: TMS-induced changes in neuronal activity can propagate through neural networks, influencing both local and distant brain regions [72]. This network-level impact involves complex interactions between neurons, glial cells, and other supporting structures. Mechanisms of action: The mechanisms through which TMS affects different cell types are multifaceted. The generation of action potentials in neurons leads to synaptic changes and the release of neurotransmitters. These neurotransmitters and changes in astrocytic activity contribute to the overall modulatory effects of TMS on brain function [19]. Understanding the cellular specificity of TMS effects on neurons, astrocytes, microglia, and pericytes is an evolving area of research to elucidate the precise molecular and cellular mechanisms underlying the impact of TMS on the brain at the cellular and network levels.

**Figure 2:**
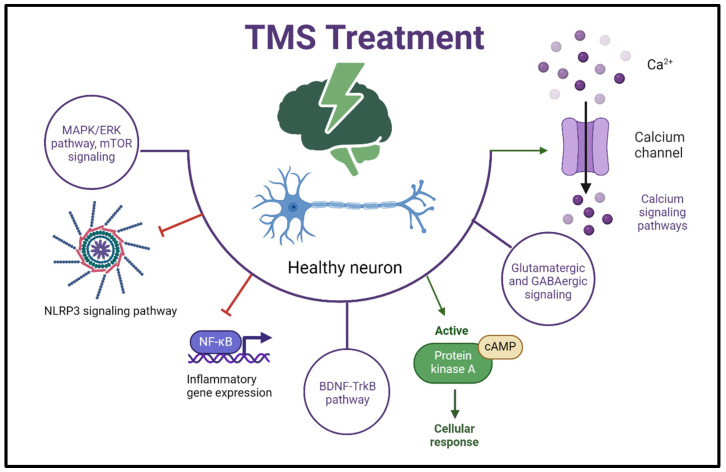
TMS affects molecular mechanisms and impacts cellular pathways. Complex interactions and signaling cascades are involved in the pathways activated by TMS in neurons. TMS alters neuronal membrane calcium ion flow and influences glutamate and GABA release. TMS activates cAMP and PKA pathways, the BDNF-TrkB pathway. TMS triggers the MAPK/ERK pathway and mTOR pathway. TMS-induced neural changes affect the inflammatory response. These pathways are crucial for TMS’s effects on neural function and behavior. Figure 2 was created with BioRender.

The cellular pathways activated by TMS involve complex interactions and signaling cascades within neural cells (Figure 2). Calcium signaling pathways: TMS induces changes in the flow of calcium ions across neuronal membranes [68]. This calcium influx is a central signaling event in neurons and can activate various downstream pathways involved in synaptic transmission, gene expression, and cellular plasticity. Glutamatergic and GABAergic signaling: TMS affects the release of neurotransmitters, particularly glutamate and gamma-aminobutyric acid (GABA) [76]. These neurotransmitters are pivotal in excitatory and inhibitory synaptic transmission, influencing cellular responses and network activity. cAMP and PKA signaling: TMS-induced neuronal depolarization can activate cyclic adenosine monophosphate (cAMP) and protein kinase A (PKA) signaling pathways [77]. These pathways are involved in intracellular signal transduction and modulation of gene expression. BDNF-TrkB pathway: Brain-derived neurotrophic factor (BDNF) is a neurotrophin associated with neuronal survival, growth, and synaptic plasticity. TMS has been shown to modulate the BDNF-TrkB pathway, influencing neuroplasticity and long-term cellular changes [73]. MAPK/ERK pathway: The mitogen-activated protein kinase/extracellular signal-regulated kinase (MAPK/ERK) pathway is implicated in cell growth, differentiation, and synaptic plasticity. TMS-induced neuronal activation may lead to the activation of this pathway [78]. mTOR Signaling: The mammalian target of the rapamycin (mTOR) pathway is involved in regulating protein synthesis and cellular homeostasis. TMS has been associated with changes in mTOR signaling [79]. Inflammatory pathways: TMS-induced changes in neural activity may have downstream effects on inflammatory pathways involving cytokines and microglial activation [80]. Neurotransmitter receptor signaling: TMS affects the release and availability of neurotransmitters, leading to the activation of downstream signaling pathways mediated by neurotransmitter receptors (e.g., glutamate receptors and GABA receptors) [12,57]. Understanding the cellular pathways that TMS activates is crucial for unraveling its effects on neural function and behavior.

## 4. Clinical Applications: TMS for ADHD and Depression

ADHD is a neurodevelopmental disorder that can affect both children and adults. It is characterized by persistent inattention and hyperactivity/impulsivity that interferes with daily functioning or development [18,81]. ADHD symptoms can be divided into two main types: inattention and hyperactivity/impulsivity. The following are common symptoms associated with ADHD: difficulty maintaining attention, easily distracted by irrelevant stimuli, poor organizational skills, and forgetfulness [82]. Impact on daily functioning: For a diagnosis of ADHD, symptoms should significantly interfere with the person’s social interactions and academic or occupational functions. Depression is a mental health condition characterized by persistent feelings of sadness, hopelessness, and a lack of interest or enjoyment in activities [83]. The symptoms of depression vary from person to person, as do the severity and duration of symptoms. The following are common symptoms associated with depression: persistent sadness, loss of interest, irritability, and social withdrawal. Cognitive symptoms include difficulty concentrating, making decisions, or remembering things [84]. Understanding the neural basis of attention is crucial for unraveling the complexities of human cognition and developing targeted interventions for attention-related challenges.

**Figure 3:**
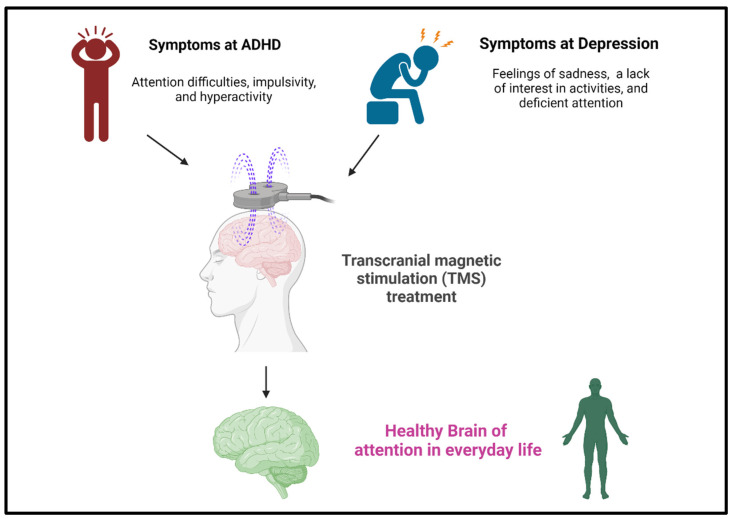
TMS is a noninvasive neuromodulation technology that uses magnetic fields to stimulate nerve cells in the brain. TMS is a therapeutic technique primarily used to treat mental illnesses such as depression, and research has also explored its potential in addressing symptoms of ADHD. Figure 3 was created with BioRender.

The efficacy of TMS in enhancing attention in healthy individuals involves the application of magnetic fields to specific areas of the brain, affecting neural activity [19]. Several studies have explored the potential of TMS to enhance cognitive functions, including attention, in healthy individuals [85,86,87]. The idea is to modulate neural circuits related to attention and executive functions to improve cognitive performance. Targeted stimulation: Researchers studied the effects of TMS on specific brain regions involved in attention, such as the dorsolateral prefrontal cortex [88,89]. The goal of delivering magnetic pulses to these areas is to enhance neural activity and potentially improve attentional processes. The idea behind using TMS to treat ADHD (Figure 3) is to modulate neural activity in areas associated with attention and impulse control, such as the prefrontal cortex. TMS treats depression (Figure 3) by delivering magnetic pulses to specific brain areas. Usually, the prefrontal cortex modulates neural activity and relieves symptoms of depression.

Given the unique nature of ADHD and depression, combining research on these disorders into a single research effort requires a solid and clear justification. Here are a few key considerations to justify this combination: ADHD and depression may present with overlapping symptoms, such as difficulty concentrating and impaired executive functioning [90,91]. Studying them together may provide insights into shared neurobiological mechanisms. Both ADHD and depression involve changes in neurotransmitter systems such as dopamine, norepinephrine, and serotonin [92,93]. Studying these common pathways may reveal shared neurological disorders. Since both disorders are associated with synaptic dysfunction, studying them together allows for a comprehensive exploration of how synaptic abnormalities lead to different cognitive and emotional symptoms. Compared with conducting separate studies, combining studies may save resources, discover common mechanisms, and optimize treatments. The rationale for using TMS to study ADHD and depression simultaneously, integrating attention research and clinical applications, is as follows [94]. Both ADHD and depression are associated with disruptions in neural circuits related to attention and executive function. Studying these disorders simultaneously through TMS can investigate shared neurobiological substrates, potentially revealing common mechanisms of attention disorders. TMS allows selective modulation of specific brain regions involved in attentional networks. Studying attentional networks using TMS in the context of ADHD and depression could explore how the disruption of these networks contributes to attentional deficits in each disorder—neurobiological implications of TMS for improving attentional understanding during depressive states. Incorporating depressive states into studies utilizing TMS provides a unique perspective for exploring attentional processes. TMS has been shown to induce neuroplastic changes in the brain, particularly in attentional processes during states of depression [95]. Research on TMS in depression often focuses on the prefrontal cortex, a critical region involved in the control of attention. Changes in prefrontal function associated with depressive states can be modulated by TMS, thereby helping to improve cognitive function [95]. TMS has been shown to modulate neurochemical activity, including neurotransmitter systems involved in attention, such as serotonin and norepinephrine. Targeting specific neurotransmitter systems through TMS intervention may provide a mechanism to optimize attentional function in individuals in depressive states [96]. In conclusion, incorporating melancholic states into TMS studies enriches our understanding of attentional processes. TMS-induced neuroplasticity, modulation of the prefrontal cortex, changes in connections within the attention network, and alterations in neurochemicals can help resolve attentional deficits and enhance cognitive function in patients with depression.

### 4.1. TMS Is an Effective Clinical Treatment for ADHD

The combined application of brain neuroimaging and TMS holds promise for the clinical treatment of ADHD by providing insights into its neural underpinnings and offering targeted interventions [97,98]. Advanced neuroimaging techniques like fMRI can identify specific brain regions and networks associated with ADHD [99,100]. These imaging findings can serve as neural markers for diagnosis and treatment planning. Personalized treatment: Neuroimaging data can guide personalized treatment plans by pinpointing areas of dysfunction. Individual differences in brain activity can inform tailored interventions. Tracking treatment progress: Neuroimaging can monitor changes in brain activity as a response to treatment. This feedback helps clinicians assess the efficacy of interventions and adjust protocols accordingly. Targeted stimulation: TMS can stimulate brain regions implicated in attention deficits, such as the prefrontal and parietal cortex [101]. By modulating these areas, TMS aims to enhance attentional control. Neuroplasticity enhancement: TMS-induced neuroplastic changes could help rewire attention networks, promoting better attentional functions over time [17]. Combining TMS with neuroimaging: Integrating TMS with neuroimaging techniques allows real-time monitoring of brain activity during stimulation sessions [20]. This integration ensures accurate targeting and assessment of TMS effects. Neural network understanding: Neuroimaging helps uncover the complex interactions between brain regions contributing to attention deficits. This understanding guides the selection of TMS target areas [12]. Neuroplasticity profiling: Neuroimaging can provide insights into the neuroplasticity profiles of individuals with ADHD [102]. This information informs the design of TMS protocols tailored to each patient’s natural characteristics. Integrating brain neuroimaging and TMS in the clinical treatment of ADHD showcases a comprehensive and personalized approach [103]. Leveraging neuroimaging insights to guide TMS interventions can offer targeted and adaptive treatments that address the underlying neural mechanisms of attention deficits [63]. The integration of brain neuroimaging and TMS in ADHD treatment is an evolving field that requires robust research and validation to establish its effectiveness.

Table 1 collectively contributes to understanding how TMS may impact neural activity and cognitive functions in individuals with ADHD, providing insights into potential therapeutic applications of TMS in this population. In examining the literature on TMS for ADHD, the studies collectively contribute to a multifaceted understanding of the potential therapeutic effects of TMS on neural functioning in individuals with ADHD. Kahl et al. (2022) delve into the neurobiological aspect by investigating differences in neurometabolic and TMS motor maps in children with ADHD, shedding light on the underlying neural mechanisms associated with the disorder [104]. Detrick et al. (2021) expand the exploration into the cognitive domain by focusing on motor cortex modulation and its interaction with reward processing in children with ADHD. This study adds a behavioral dimension, examining how TMS may influence motor functions and the reward system, a critical aspect of ADHD [105]. Chen et al.’s meta-analysis (2023) provides a comprehensive overview of existing research, synthesizing evidence on the therapeutic efficacy of repetitive TMS for cognitive functions in ADHD. This meta-analytical approach enhances the generalizability of findings and offers a broader perspective on the cumulative impact of TMS interventions [106]. Bleich-Cohen et al. (2021) contribute valuable insights through using fMRI to evaluate the efficacy of prefrontal cortex deep TMS in adults with ADHD. This neuroimaging approach provides a nuanced understanding of the neural changes associated with TMS treatment in adults [58]. Weaver et al.’s pilot study (2012) takes a step toward clinical application by investigating TMS’s feasibility and potential effectiveness in treating ADHD in adolescents and young adults. This practical aspect of the research is crucial for translating scientific findings into real-world therapeutic interventions [107]. In summary, the amalgamation of these studies offers a comprehensive exploration of TMS’s neurobiological, cognitive, and clinical dimensions as a potential intervention for ADHD. The diverse methodologies employed across these studies collectively contribute to a more holistic understanding of the intricate relationship between TMS and ADHD across different age groups, paving the way for future research and potential therapeutic applications.

### 4.2. TMS Technology Is an Effective Method for Regulating Depression

Both brain neuroimaging and TMS can be applied to help regulate attention and manage emotions in people with depression [108,109]. Here is how these techniques can contribute to depression therapy. Brain neuroimaging: Identifying dysregulated networks with neuroimaging can reveal altered brain networks associated with attention deficits and emotional dysregulation in individuals with depression [110]. This understanding guides the selection of targeted interventions. Attentional bias assessment: Neuroimaging can detect attentional biases towards negative stimuli in depression [111]. This information informs strategies to redirect attention away from negative cues and promote more balanced attentional processing. Neural correlates of emotion regulation: Neuroimaging can identify brain regions involved in emotion regulation [112]. This insight helps design interventions that enhance the brain’s ability to manage and modulate emotional responses. TMS: TMS to the prefrontal cortex can modulate mood-related circuits and improve executive functions, including attention regulation and emotion management [113]. Targeted emotion-related brain regions: TMS can be applied to brain regions like the amygdala or insula, which play roles in emotion processing. By modulating activity in these areas, TMS aims to influence emotional responses and regulation [114]. Neuroplasticity effects on emotion regulation: TMS-induced neuroplastic changes can potentially reshape emotional processing circuits, leading to better emotion regulation over time [72]. The application of brain neuroimaging and TMS in depression therapy offers a multifaceted approach to addressing attention regulation and emotion management [25]. Leveraging insights from neuroimaging to guide TMS interventions can create personalized treatment strategies that directly target the neural mechanisms underlying attentional deficits and emotional dysregulation [115].

Table 2 presents literature comprises a diverse array of studies investigating the therapeutic potential of TMS for depression. These studies collectively contribute to our understanding of TMS as a multifaceted intervention for depression, encompassing various aspects of its application and efficacy. The THREE-D trial by Blumberger et al. (2018) is particularly significant for comparing different TMS modalities, shedding light on the potential noninferiority of theta burst stimulation in treating depression [116]. Meanwhile, Yu et al. (2022) add a cognitive perspective by investigating the impact of repetitive TMS on response inhibition, providing insights into the broader cognitive effects of TMS in individuals with major depression [117]. Fitzgerald’s (2021) critical analysis raises important questions about the precision of TMS targeting in depression, emphasizing the need for a deeper understanding of the neural circuits being stimulated and the optimization of stimulation parameters [118]. Kaster et al.’s (2023) exploration of symptom cluster responses to TMS treatment offers a nuanced approach to tailoring interventions based on specific symptomatology, potentially paving the way for more personalized treatment strategies [119]. The study by Schaffer et al. (2021) addressing low-frequency TMS in depressed individuals with impaired cognitive functioning represents a specialized investigation into a subgroup of patients who may present unique challenges and opportunities for treatment [120]. Lastly, Yıldız et al.’s (2023) randomized controlled study on cognitive outcomes in treatment-resistant depression provides valuable insights into the broader cognitive effects of TMS interventions in a particularly challenging population [121]. In summary, these studies collectively contribute a rich tapestry of insights into the effectiveness, cognitive impacts, targeting precision, and tailored responses of transcranial magnetic stimulation in depression. This evolving body of research highlights the potential versatility of TMS as a therapeutic tool and underscores the importance of continued exploration to refine its application and maximize its benefits for individuals with depression.

### 4.3. Linking Synaptopathies to Synaptic Plasticity in Neurological Disorders and TMS: A Focus on ADHD and Depression

Incorporation of relevant findings on the basic biological principles of synaptic plasticity, including the Hebbian theory of synaptic plasticity, a concept that emphasizes its role in learning, memory, and adaptive changes in neural circuits [122], explains how synaptic connections between neurons are strengthened, emphasizing the principle that “cells that fire together connect” [123]. The presynaptic neuron is consistently active, and the activation of a postsynaptic neuron closely follows its activity; the synaptic connection between them will be strengthened. Mechanism of strengthening: When a presynaptic neuron’s action potential repeatedly triggers the firing of a postsynaptic neuron, the synaptic strength increases. This strengthening is believed to result from structural or functional changes at the synapse. Neural circuit plasticity: Hebbian plasticity is a form of activity-dependent synaptic plasticity [124]. It is a mechanism by which neural circuits can adapt and change based on experience. Application to memory: Hebbian plasticity is often invoked to explain the cellular and molecular mechanisms underlying the formation and maintenance of memories. It provides a model for reinforcing synaptic connections associated with specific experiences. Synaptic consolidation: Hebbian plasticity is part of the broader process of synaptic consolidation, where recently acquired information is stabilized in neural circuits by strengthening synaptic connections [125]. LTP and long-term depression (LTD): The two primary forms of synaptic plasticity are LTP and LTD, where LTP involves the strengthening of synaptic connections, while LTD involves synaptic weakening. Both processes play crucial roles in shaping neural circuits and are often studied in the context of learning and memory. Molecular mechanisms of synaptic plasticity: The molecular mechanisms underlying synaptic plasticity are briefly outlined. The involvement of neurotransmitter receptors, particularly NMDA receptors, and intracellular signaling pathways, such as those involving calcium and various kinases, are highlighted [126]. In summary, Hebbian theory explains how synaptic connections are modified based on the relative activity of neurons. This principle has broad implications for our understanding of learning, memory, and neuroplasticity, and it forms the basis for studying how synaptic changes affect various cognitive functions and neurological diseases.

Neuropsychiatric disorders such as ADHD and depression are complex disorders with multifaceted pathophysiological underpinnings. One aspect is the role of synaptopathies, disorders of synaptic transmission, and the development and manifestation of these disorders. Synaptopathies in ADHD and depression involve the pathophysiology of ADHD and depression, with a focus on synaptopathies [127,128]. Synaptopathies involve dysfunction in synaptic transmission and are implicated in various neuropsychiatric disorders. Synaptopathies and impaired synaptic plasticity in ADHD: ADHD, characterized by attention deficits and impulsivity, is associated with disruptions in synaptic plasticity within crucial brain regions [129]. Studies suggest that the dysregulation of neurotransmitters involved in synaptic plasticity, such as dopamine and glutamate, contributes to altered connectivity and impaired long-term potentiation. In depression, a mood disorder marked by persistent negative affect, synaptic plasticity is perturbed in circuits associated with mood regulation. Changes in the expression of neurotrophic factors and alterations in the density of synaptic connections may contribute to the impaired synaptic plasticity observed in depression [130]. The role of synaptic plasticity in symptom expression: Disruptions in synaptic plasticity have profound implications for the expression of symptoms in both ADHD and depression [131,132]. In ADHD, impaired synaptic plasticity may underlie difficulties in learning, attention, and impulse control. In depression, alterations in synaptic plasticity could contribute to the persistence of negative emotional states and the reduced ability to experience pleasure. In summary, the intricate dance between synaptopathies and synaptic plasticity is a critical player in the pathophysiology of neurological disorders, particularly ADHD and depression. Targeting mechanisms that restore or modulate synaptic plasticity may offer novel treatment approaches for ADHD and depression.

Disruptions in synaptic transmission and plasticity characterize synaptopathies and provide a comprehensive framework for understanding the pathophysiology of neurological diseases. By influencing synaptic function, TMS may alleviate symptoms associated with disrupted neurotransmission and plasticity [72]. Based on this, the use of synaptopathy and TMS as neurostimulation techniques may have a positive impact on the symptoms of ADHD and depression. In ADHD, where synaptic dysregulation is implicated, TMS emerges as a potential modulator of synaptic function [58]. By generating rapidly changing magnetic fields, TMS induces electrical currents in neurons, affecting synaptic activity. The interaction between TMS and synaptopathies may lead to neuromodulatory effects, influencing neurotransmitter release and altering synaptic plasticity. In ADHD, TMS could modulate neural circuits involved in attention and impulse control, addressing the underlying synaptopathies [133]. In depression, characterized by disrupted synaptic plasticity, TMS has demonstrated therapeutic potential. TMS, particularly rTMS, modulates neuronal activity and synaptic plasticity in mood-regulating circuits [134]. The magnetic fields generated by TMS may influence neurotransmitter release and receptor activity, thereby impacting synaptic communication. The synergy between TMS and synaptopathies potentially contributes to the observed antidepressant effects, offering a neurostimulatory approach to correct synaptic abnormalities in depression. In depression, the neuromodulatory effects of TMS may promote synaptic plasticity in mood-regulating circuits, contributing to the amelioration of depressive symptoms [25]. Exploring the specific synaptic mechanisms affected by TMS in ADHD and depression may guide the development of targeted stimulation protocols. The convergence of synaptopathies, TMS findings, and neurological disorders provides a nuanced perspective on the interaction of neurostimulation techniques with synaptic function. Unraveling the complex relationship between TMS and synaptopathies could enhance understanding of the effects observed in ADHD and depression.

## 5. Conclusions

In conclusion, the ever-evolving fields of attentional neuroimaging and TMS have provided significant advancements that deepen our understanding of the intricate mechanisms underlying attention [135]. The convergence of these two domains offers a multifaceted perspective on attentional processes, from their neural underpinnings to their potential modulation for cognitive enhancement and clinical interventions [136]. Neuroimaging techniques, such as fMRI, EEG, and MEG, have unveiled the dynamic interplay of brain regions and networks involved in attention [18,137,138,139]. These methods have enabled the mapping of attention networks, revealing the complexity of interactions among regions like the prefrontal cortex, parietal cortex, and thalamus [140]. Connectivity analyses have illuminated the relationships and communication pathways that govern attentional functions. Furthermore, integrating multiple modalities through multimodal fusion has enriched our understanding by providing a comprehensive view of attentional processes.

TMS interactions with synaptic function in ADHD are paths to restoration. ADHD is intricately linked to disruptions in synaptic function, creating challenges in attention, impulse control, and hyperactivity. TMS, as a neurostimulation technique, emerges as a promising avenue to interact with synaptic function and address the underlying synaptopathies in ADHD [133]. Through its ability to induce electrical currents in neurons, TMS can modulate synaptic function in ADHD-relevant brain regions [106]. TMS’s rapidly changing magnetic fields may influence neurotransmitter release and alter the balance of excitatory and inhibitory signals within neural circuits. By targeting specific areas implicated in ADHD pathophysiology, TMS provides a noninvasive means to interact with synaptic function at a neurobiological level. The interaction between TMS and synaptic function holds the potential to address the synaptopathies associated with ADHD. By modulating the activity of neurons and influencing neurotransmitter systems, TMS may contribute to restoring optimal synaptic communication. By explicitly targeting regions associated with ADHD pathophysiology, TMS could induce neuroplastic changes, promoting synaptic plasticity and optimizing the connectivity within affected neural circuits [101]. This synaptic restoration approach has the potential to address the root causes of ADHD symptoms rather than merely alleviating their manifestations. In conclusion, TMS, as a neurostimulation technique, presents a unique opportunity to interact with synaptic function and contribute to observed effects in ADHD. TMS interventions may be crucial in restoring optimal synaptic communication by directly influencing neuronal activity and modulating neurotransmitter systems.

Depression is intricately linked to synaptic dysfunction, involving alterations in neurotransmitter release, receptor activity, and synaptic plasticity. As a neurostimulation technique, TMS offers a nuanced approach to interact with synaptic function, providing insights into the neurobiology underlying its observed effects on depression [141]. The modulation of neural networks contributes to the observed antidepressant effects of TMS. TMS exerts its impact on synaptic function by influencing neurotransmitter systems crucial in depression, such as serotonin, norepinephrine, and dopamine [142]. The magnetic fields generated by TMS may alter the release and reuptake of these neurotransmitters, restoring a balance often disrupted in depressive states [96]. By directly impacting synaptic transmission, TMS plays a role in recalibrating the neurochemical milieu associated with depression. At the core of TMS-induced changes lies synaptic plasticity—the brain’s ability to adapt and reorganize. TMS, especially high-frequency rTMS, has been shown to promote LTP and induce changes in synaptic strength [143]. The modulation of synaptic plasticity becomes a pivotal mechanism through which TMS interacts with the intricate synaptic networks affected in depression. In conclusion, the neurobiology of TMS intervention in depression involves a multifaceted interaction with synaptic function.

On the other hand, TMS has emerged as a powerful tool to explore and modulate attentional circuits [144]. The application of TMS at specific frequencies and targeted brain regions has led to plastic changes that optimize attention-related neural pathways [72]. The ability to temporarily enhance or inhibit specific brain regions through TMS has illuminated the causal relationships between brain activity and attentional functions [12,145]. Repeated TMS sessions and personalized protocols promise to extend these effects and offer individualized attentional interventions [146]. The synergy between attentional neuroimaging and TMS shapes the landscape of attention research [147]. By integrating the insights from neuroimaging with the manipulative capabilities of TMS, researchers are deciphering the causal mechanisms that underlie attention and exploring new avenues for enhancing attentional processes. This multidisciplinary approach has far-reaching implications, from identifying the neural correlates of attention states to designing personalized interventions for attention-related disorders [148]. As these fields advance, the potential for translational applications becomes increasingly evident. The integration of these techniques holds promise for optimizing cognitive functions in healthy individuals, improving attention-related deficits in clinical populations, and tailoring interventions to individual cognitive profiles [149]. This synergy ultimately contributes to a more nuanced and comprehensive understanding of attention, paving the way for innovative strategies to enhance attentional mechanisms and their impact on daily life.

Indeed, further studying the application of attention improvement techniques in clinical settings and daily life contexts is paramount. The implications of such research are substantial and can potentially transform the lives of individuals with attention-related challenges and those seeking to optimize their cognitive performance [91]. Attention is intertwined with mental well-being. Investigating how attention improvement techniques impact psychological states and emotional regulation can inform their potential role in promoting mental health [150]. In summary, studying the application of attention improvement interventions in clinical and daily life contexts is a pivotal step toward translating scientific advancements into practical solutions. Such research has the potential to bridge the gap between theory and real-world impact, ultimately benefiting individuals with attention-related challenges and enhancing cognitive performance across various domains.

## Figures and Tables

**Table 1:** ADHD is a neurodevelopmental disorder characterized by inattention, hyperactivity, and impulsivity symptoms. There is some evidence to suggest that TMS may be beneficial in reducing symptoms of ADHD.

Study	Effects	Reference
Kahl, C. K. et al. (2022)	This study analyzed differences in neurometabolic and cortical motor representations obtained through TMS in 26 children with ADHD and 25 typically developing children. Furthermore, detected changes in neurometabolite levels (glutamate, glutamine, and GABA) correlated with TMS-derived measurements. Research has found that the neurochemistry and neurophysiology of critical nodes in the motor network may be altered in children diagnosed with ADHD.	[104]
Detrick, J. A. et al. (2021)	Fifty-five children diagnosed with ADHD, of whom thirty-seven were male, and fifty typically developing control children between the ages of 8 and 12 years, of whom thirty-two were male, took part in the research—a child-friendly reward-motivated task evaluated cortical disinhibition with noninvasive TMS. The significant results revealed modifications in short-interval cortical inhibition and increased the amplitudes of motor-evoked potentials obtained during the reward task. Overall, this research endorses the potential utility of transcranial magnetic stimulation-induced cortical inhibition and task-evoked excitability as biomarkers for areas of clinically significant dysfunction in childhood ADHD.	[105]
Chen, Y. H. et al. (2023)	This meta-analysis of 189 participants (average age in the adult and child/adolescent groups was 32.78 and 8.53 years, respectively) showed that rTMS was more effective than a control group in improving persistence. Results support the therapeutic efficacy of rTMS in enhancing sustained attention and processing speed in ADHD patients.	[106]
Bleich-Cohen, M. et al. (2021)	This study investigated the neural effects of high-frequency repetitive deep TMS applied to the right or left PFC in 62 adults with ADHD. Increased activation in the right dorsolateral prefrontal cortex (rDLPFC), right parietal cortex, and right insula/inferior frontal gyrus (IFG) were found after stimulation treatment. This study shows that dTMS can effectively modulate attention-related brain networks, suggesting it is a viable technique that can improve attention symptoms in adults with ADHD.	[58]
Weaver, L. et al. (2012)	TMS was applied to the right prefrontal cortex at 10 Hz (100% of the observed motor threshold) for 2000 pulses each. The study used a sham-controlled crossover design with nine participants. The ten sessions of TMS lasted over two weeks, with a one-week interval between active and sham phases. In summary, TMS is safe and effective in treating ADHD in adolescents and young adults.	[107]

**Table 2:** Depression is a significant mental health disorder that typically manifests with symptoms such as persistent low mood and reduced attention. Some evidence suggests that TMS may be beneficial in improving depression.

Study	Effects	Reference
Blumberger, D. M. et al. (2018)	Efficacy, safety, and tolerability of standard high-frequency (10 Hz) rTMS in adults with treatment-resistant depression. Participants were aged 18–65 years and diagnosed with treatment-resistant depression and major depressive disorder. Two hundred five participants were assigned to the 10 Hz rTMS group. The 10 Hz rTMS treatment approach was safe and tolerable.	[116]
Yu, F. et al. (2022)	People with major depressive disorder (MDD) often exhibit cognitive impairment. This study investigated the effects of individualized rTMS targeting the left dorsolateral prefrontal cortex (lDLPFC)–nucleus accumbens (NAcc) network in patients with major depressive disorder (MDD). In a double-blind, sham-controlled trial, 44 patients diagnosed with MDD were randomly assigned to receive active rTMS (10 Hz, 100% of resting motor threshold) or sham rTMS. Research shows that individualized rTMS treatment may be a practical approach for patients with depression.	[117]
Fitzgerald, P. B. et al. (2021)	The use of rTMS in treating depression highlights the importance of improved local stimulation methods. It is recommended that optimal results from rTMS be achieved by stimulating relatively anterior parts of the left DLPFC. rTMS is an established treatment for patients with depression who do not respond well to antidepressant medications. Although effective, there is room for improvement in clinical outcomes.	[118]
Kaster, T. S. et al. (2023)	A study to examine differential responses of symptom clusters to rTMS in patients with treatment-resistant depression (TRD). rTMS is a treatment for depression that targets specific neural circuits, and these trials involved delivering rTMS to the left DLPFC. The study included 596 participants with TRD; preliminary analysis of the THREE-D treatment trial shows that symptom clusters differ in response to rTMS treatment. The anxiety symptom cluster was significantly less responsive to treatment than the other symptom clusters. In summary, this study highlights the potential to tailor rTMS treatment to the specific symptom clusters experienced by patients with treatment-resistant depression, with a focus on addressing the differential responses of these symptom clusters to rTMS treatment.	[119]
Schaffer, D. R. et al. (2021)	A study exploring the use of low-frequency TMS (LF-TMS) in the treatment of depression in cognitively impaired individuals. Fifty-three participants received LF-TMS treatment. LF-TMS resulted in significant reductions in individual depressive symptoms in both cognitive function groups. The significant group-by-time interaction showed differential effects between the two cognitive function groups. In summary, this study suggests that LF-TMS may be a promising treatment for depressive symptoms in individuals with impaired cognitive function, with potential additional benefits on neurocognitive function.	[120]
Yıldız, T. et al. (2023)	This study included 30 patients with depression (aged 18–50 years) to explore the potential benefits of TMS on cognitive function in patients with treatment-resistant depression. TMS may be beneficial in improving cognitive function in patients with treatment-resistant depression and may provide early cognitive improvements during treatment.	[121]

## Data Availability

Not applicable.
